# 99. Presence of Chronic Diseases and Compliance with Québec Provincial Guidelines for Outpatient Antibiotic Prescription, Québec, Canada, 2010-2017

**DOI:** 10.1093/ofid/ofab466.301

**Published:** 2021-12-04

**Authors:** Elise Fortin, Geneviève Deceuninck, Caroline Sirois, Caroline Quach, Marc Simard, Sonia Jean, Alejandra Irace-Cima, Nadine Magali-Ufitinema

**Affiliations:** 1 Institut national de santé public du Quebec, Quebec, Quebec, Canada; 2 Institut national de santé publique du Québec, Quebec, Quebec, Canada; 3 Université Laval, Quebec, Quebec, Canada; 4 Montreal University, Montreal, Quebec, Canada; 5 Ministère de la Santé et des Services sociaux, Montreal, Quebec, Canada

## Abstract

**Background:**

In Québec primary care, antimicrobial use is higher in patients with chronic diseases, but it is unclear whether this utilization may be reduced. We aimed to measure the proportion of compliant antimicrobial prescriptions according to the provincial guidelines for the treatment of common respiratory and urinary infections and measure variations in this proportion with certain chronic diseases.

**Methods:**

Antimicrobial dispensing covered by the public drug insurance plan between April 2010 and March 2017, delivered within 2 days of an outpatient consultation for an infection was included. Infections targeted by provincial guidelines were studied: otitis media, pharyngitis, pneumonia, sinusitis, bronchitis and chronic obstructive pulmonary disease exacerbations, cystitis, and acute pyelonephritis. The proportion of prescriptions compliant with guidelines (right antimicrobial for children, and right antimicrobial and dosage for adults) was computed by age group (children or adults) and per category of chronic disease (respiratory, cardiovascular, diabetes, mental disorder, none of previous). For each infection and age group, multivariate robust Poisson regression was used to measure the impact of categories of chronic diseases on proportions of prescriptions compliant with guidelines.

**Results:**

Between 14 677 and 312 786 prescriptions were included, for each infection. Compliance to guidelines was above 87% in children and was significantly lower (≤ 3% bellow) in children with asthma. In adults, the choice of agent was compliant for at least 73% of prescriptions, except for cases of pharyngitis (between 53% and 61%). Accounting for dosage led to lower proportions of compliance, which varied between 19% (cystitis with diabetes) and 77% (pyelonephritis with none of the studied chronic disease categories). Compliant prescriptions were 2,4% to 20,4% less frequent in the presence of chronic diseases (statistically significant).

**Conclusion:**

Non-compliant prescriptions could still be appropriate, but their high frequency suggests there is room for improvement. Dosage seems particularly problematic. Additional support could be offered to clinicians for the prescription of antimicrobials to patients with chronic diseases.

**Disclosures:**

**All Authors**: No reported disclosures

